# Effects of Habitat River Microbiome on the Symbiotic Microbiota and Multi-Organ Gene Expression of Captive-Bred Chinese Giant Salamander

**DOI:** 10.3389/fmicb.2022.884880

**Published:** 2022-06-13

**Authors:** Wei Zhu, Chunlin Zhao, Jianyi Feng, Jiang Chang, Wenbo Zhu, Liming Chang, Jiongyu Liu, Feng Xie, Cheng Li, Jianping Jiang, Tian Zhao

**Affiliations:** ^1^Chinese Academy of Sciences (CAS) Key Laboratory of Mountain Ecological Restoration and Bioresource Utilization & Ecological Restoration Biodiversity Conservation Key Laboratory of Sichuan Province, Chengdu Institute of Biology, Chengdu, China; ^2^Key Laboratory of Bioresources and Ecoenvironment (Ministry of Education), College of Life Sciences, Sichuan University, Chengdu, China; ^3^State Key Laboratory of Environmental Criteria and Risk Assessment, Chinese Research Academy of Environmental Sciences, Beijing, China

**Keywords:** amphibian, conservation, immunity, microbiome, pathogen, reintroduction

## Abstract

The reintroduction of captive-bred individuals is a primary approach to rebuild the wild populations of the Chinese giant salamander (*Andrias davidianus*), the largest extant amphibian species. However, the complexity of the wild habitat (e.g., diverse microorganisms and potential pathogens) potentially threatens the survival of reintroduced individuals. In this study, fresh (i.e., containing environmental microbiota) or sterilized river sediments (120°C sterilized treatment) were added to the artificial habitats to treat the larvae of the Chinese giant salamander (control group—Cnt: 20 individuals, treatment group 1 with fresh river sediments—T1: 20 individuals, and treatment group 2 with sterilized river sediments—T2: 20 individuals). The main objective of this study was to test whether this procedure could provoke their wild adaptability from the perspective of commensal microbiotas (skin, oral cavity, stomach, and gut) and larvae transcriptomes (skin, spleen, liver, and brain). Our results indicated that the presence of habitat sediments (whether fresh or sterilized) reshaped the oral bacterial community composition. Specifically, Firmicutes decreased dramatically from ~70% to ~20–25% (mainly contributed by *Lactobacillaceae*), while Proteobacteria increased from ~6% to ~31–36% (mainly contributed by Gammaproteobacteria). Consequently, the proportion of antifungal operational taxonomic units (OTUs) increased, and the function of oral microbiota likely shifted from growth-promoting to pathogen defense. Interestingly, the skin microbiota, rather than the colonization of habitat microbiota, was the major source of the pre-treated oral microbiota. From the host perspective, the transcriptomes of all four organs were changed for treated individuals. Specifically, the proteolysis and apoptosis in the skin were promoted, and the transcription of immune genes was activated in the skin, spleen, and liver. Importantly, more robust immune activation was detected in individuals treated with sterilized sediments. These results suggested that the pathogen defense of captive-bred individuals was improved after being treated, which may benefit their survival in the wild. Taken together, our results suggested that the pre-exposure of captive-bred Chinese giant salamander individuals to habitat sediments could be considered and added into the reintroduction processes to help them better adapt to wild conditions.

## Introduction

Amphibian is one of the most threatened taxa on Earth today, with approximately one-third (32%) of the wild populations declining worldwide due to global changes caused by human activities (Stuart et al., 2004; González-Del-Pliego et al., 2019). Therefore, amphibian conservation has been advocated and suggested to be more urgent by ecologists and biologists (WWF, 2020). The reintroduction of captive-bred individuals to the wild is one of the most important measures for the rebuilding of natural amphibian populations. Typically, the artificial habitats of the captive individuals are designed to be suitable environments for them to grow (e.g., stable habitat structure, clean food, and water). Therefore, once being released to the wild, the complicated environmental condition may induce a low survival rate of captive-bred animals. Accordingly, determining how to increase the released individuals' survival rate and fitness should be the priority for species conservation.

Prerelease training is a common approach to teach captive-bred animals to prey and live in the wild (e.g., giant panda *Ailuropoda melanoleuca*; Wei et al., 2015, and Crested Ibis *Nipponia nippon*; Nagata and Yamagishi, 2016). Regarding amphibians, due to their thin, moist, and permeable skins, they are more vulnerable to the effects of desiccation, toxic chemicals (Mcmenamin et al., 2008; Wang X. et al., 2019; Zhu et al., 2022), and in particular, environmental pathogens (O'hanlon et al., 2018; Fisher and Garner, 2020). Previous studies have demonstrated that symbiotic microbes (i.e., skin, oral cavity, stomach, and gut microbes) played important roles in regulating amphibian capacity for environmental adaptation (Chang et al., 2016; Guo et al., 2021). Moreover, the composition of symbiotic microbiota is associated with host susceptibility and the composition of environmental pathogens (Rebollar et al., 2016; Bletz et al., 2017). Therefore, the reorganization of symbiotic microbiota is a potential strategy for amphibian to cope with the colonization of the host by pathogens (Muletz et al., 2012; Longo and Zamudio, 2017). Accordingly, symbiotic microbes were important for amphibian disease immunity (Hopkins, 2007; Harris et al., 2009). Different microbiota community structures were correlated with the specific immune status of the host, maintaining the mutualistic nature of the host-microbial relationship (Hooper et al., 2012). Consequently, we argued that the diversity and community structure of the symbiotic microbiota may be an important factor influencing the fitness and survival of released captive-bred individuals.

Previous studies have indicated that symbiotic microbes can shift with the change in their natural habitat (Bletz et al., 2016; Bird et al., 2018; Muletz et al., 2018), and increasing attention has been paid to understand how the symbiotic microbiota responds to these environmental changes and the implications to the host fitness (Ellison et al., 2019; Barnes et al., 2021). For example, reduced microbiome stability stemming from habitat alterations could compromise the population health of amphibians, even in the absence of pathogen infection (Neely et al., 2021). Constant exposure to the environment with complex microbiota would shape the physiological development and evolution of animals. However, since captive-bred animals usually exhibited low diversity of symbiotic microbiota (Kueneman et al., 2016; San et al., 2021), it is not clear how their health can be affected by the wild habitat with complex environmental microbiota (Hyde et al., 2016). Adding soil collected from their natural environment into the captive environment seems to be an applicable approach to rescue this situation (Loudon et al., 2014). It has been reported that amphibians could recruit putatively beneficial bacteria to defend themselves from fungal infection (Longo and Zamudio, 2017). Pre-exposure to the natural microbiota might enable an opportunity for amphibian to reorganize their microbiota and thus reinforce their immunity to potential pathogens.

The Chinese giant salamander (*Andrias davidianus*) is a flagship species for amphibian conservation. It is the largest extant amphibian species in the world, and its evolutionary history can be traced back to 16 Ma (Fei et al., 2009; Yan et al., 2018). Recent studies showed that this species was comprised of at least seven lineages, thus it should be considered as *Andrias* species in China (Yan et al., 2018; Liang et al., 2019; Turvey et al., 2019). Historically, *Andrias* species were widely distributed in central and southern China throughout the Yangtze, Yellow, and Pearl River drainages (Pierson et al., 2014; Wang et al., 2017). However, due to the dramatic decline of the wild populations (over 80%) in the past 60 years (Wang et al., 2017; Turvey et al., 2019), the distribution of *Andrias* species was concentrated in 12 patches in China currently (IUCN, 2016). These declines are largely due to habitat degradation, water pollution, and in particular, overexploitation (Liang et al., 2004; Zhao et al., 2020). Consequently, *Andrias* species were evaluated as critically endangered in the International Union for Conservation of Nature Red List (IUCN, 2016), and also in China (Jiang et al., 2016). Accordingly, increasing attention has been paid to *Andrias* species conservation by the Chinese government and national and international organizations (Pierson et al., 2014). Specifically, 47 natural reserves have been established in China to protect *Andrias* species since 1980, covering more than 240,000 km^2^ of the total area (Zhao et al., 2017). Another important protective measure for *Andrias* species conservation is the reintroduction of captive-bred individuals, and the first activity was conducted in Zhangjiajie National Nature Reserve in 2002 (China Aquatic Wildlife Conservation, 2015). Based on the records, a total of more than 287,000 captive-bred *Andrias davidianus* individuals were released into natural reserves by the end of 2019 (Shu et al., 2021). Despite a large number of individuals have been released, it is still rare to observe *Andrias* species in the field. The physiological status of captive-bred salamander may be maladaptive to the wild environment. With the increasing attentions on the potential role of symbiotic microbiota in conservation biology, the Chinese giant salamander is a great model to examine the potential effects of habitat microbiota exposure on the symbiotic microbiota and host status.

In this study, we used experimental approaches to (1) test whether pre-treatment (i.e., the pre-exposure to the field habitat sediments) altered captive-bred *Andrias* individuals in the skin, oral cavity, stomach, and gut microbe composition and (2) verify whether the change in the composition of the microbiota was related to the host physiological response (e.g., upregulation of the expression of the immune-related genes). We predicted that differentiation in the skin, oral cavity, stomach, and gut microbiota, as well as the physiological traits, can be detected between treated and controlled *Andrias* individuals. We hope our study can provide a potential approach that can activate the innate immune system of captive-bred *Andrias* individuals before releasing them back to the field. More importantly, we also hope it can increase the fitness-related traits or indices of the released individuals and thus improve the conservation strategies of *Andrias* species.

## Materials and Methods

### Animal Culture and Treatment

Sixty *A. davidianus* larvae from the same clutch were collected ~350 days after hatching (d.a.h) from a hatchery in Hongya County of Sichuan Province, China. These larvae were then transferred to Chengdu Institute of Biology, Chinese Academy of Sciences on 20 September 2019. They were acclimated in an artificial climate chest (BIC-250, Shanghai Boxun, China), raised in two plastic containers (29 × 20 × 9.7 cm), with each of them containing 30 individuals and 4 L aerated water. The artificial climate chest was kept under controlled temperature (15 ± 0.5°C) and a 12-h light/dark condition. Larvae were overfed with red worms (larvae of *Chironomus* sp.) every day at 9:00 a.m. Water in the containers was renewed entirely at 18:00 every day. The acclimation lasted for 20 days, until the start of the treatment (10 October 2019). Sterilized tap water was used as a water source in our experiment. The animal use protocol in this study (permit: 2016-AR-JJP-02) was reviewed and approved by the Animal Ethical and Welfare Committee of Chengdu Institute of Biology, Chinese Academy of Science, China. Chengdu 610041, China.

Given the demersal habit of *Andrias* individuals, we intended to create an environment with field microbiota by introducing river bottom sediments from reintroduction sites into containers containing aerated water. These sediments were convenient to be stored in large quantities in the laboratory. Therefore, sediments of Tongma River (Dujiangyan City, Sichuan Province, China; 103°38′-103°39′E, 31°02′N) were collected. We selected this river as it was an empirical habitat for the reintroduction of captive-bred *A. davidianus* since 2017. Specifically, six sites (i.e., two sites each at upstream, midstream, and downstream locations, respectively, Supplementary Figure 1) were randomly selected to collect river sediments using a mud dredger (TC-600BD-1/40, China), spanning 1.5 km of the river segment. In total, we collected ~30 kg of sediments (i.e., 5 kg per site) in the field. Approximately 50 g of the sediments from each site were separately stored in poly zipper bags for environmental microbiome analyses, and the rest sediments were mixed. All of them were preserved in a cool box in the field and were subsequently brought back to the laboratory. Sediments in the zipper bags were −80°C preserved in the freezer, which were used for analyzing microbial diversity. The mixed sediments were divided into two parts, with one part being sterilized at 120°C for 20 min in a steam sterilizer. Later, sterilized and fresh sediments were divided into 40 small packets preserved in gauzes (~300 g for each), separately, which were stored at 4°C in the fridge before utilization. For unsterilized sediments, the storage duration should not exceed 7 days, otherwise, fresh sediments were collected.

**Figure 1:**
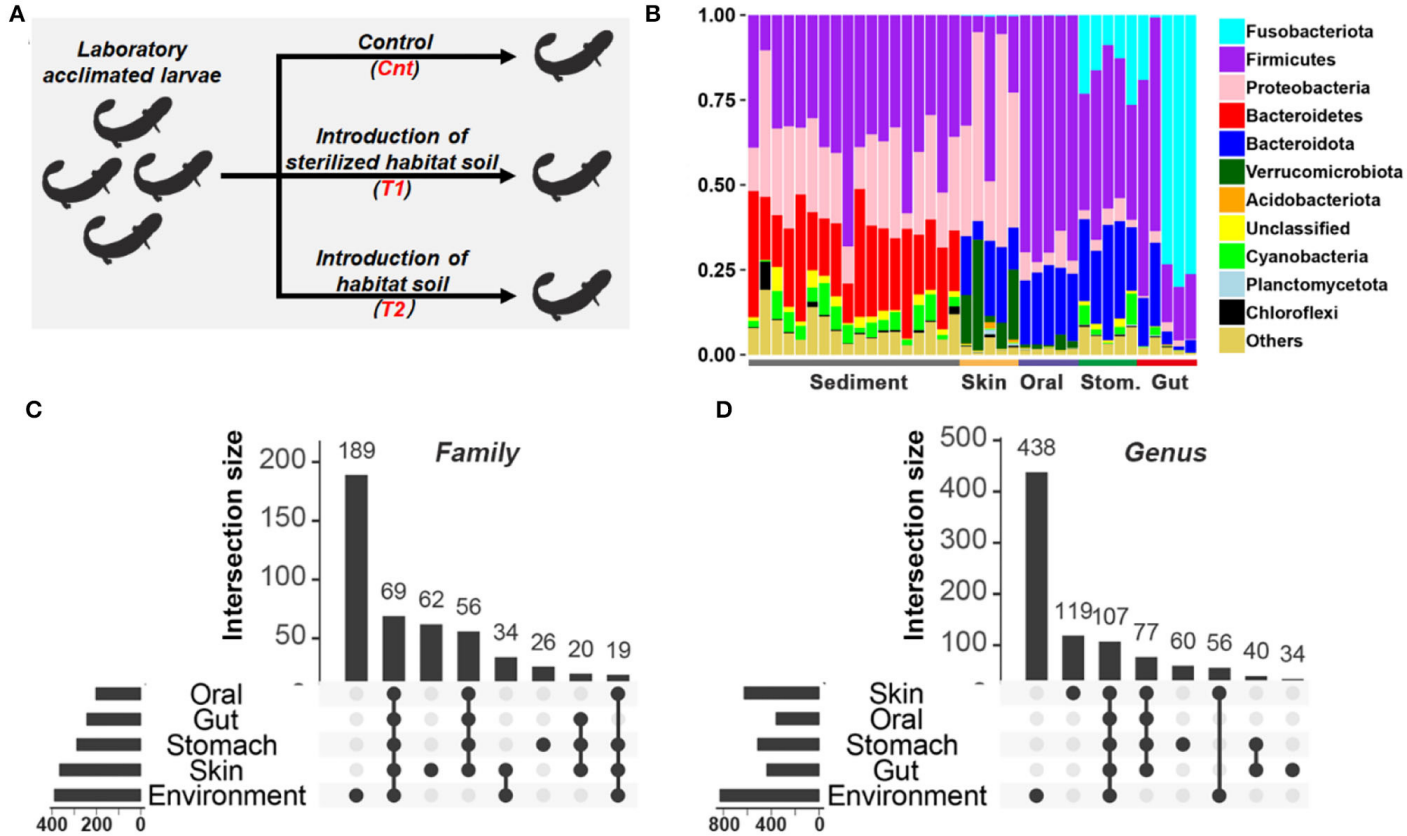
Comparison of microbiota between habitat river sediments and captive giant salamander. **(A)** Experimental design. **(B)** The relative abundance of the bacterial taxon in the habitat river sediments and captive giant salamander at the phylum level. **(C,D)** The number of shared and unique bacterial families **(C)** or genera **(D)** between river sediments and commensal microbiota. The horizontal bars represent the family or genus numbers of each source, while the vertical bars denote the numbers of their shared or unique bacterial families/genera.

Before treatment, 60 *A. davidianus* larvae were measured for the body length to the nearest 0.01 mm by a digital caliper, weighted to the nearest 0.01 g by a scale, and were then randomly divided into three groups (20 individuals per group). For each group, the 20 individuals were randomly delivered into four plastic containers (29 × 20 × 9.7 cm), with five individuals for each. Specifically, the control group (Cnt) contained only 3,000 ml of aerated water in the plastic containers, the T1 group contained 3,000 ml of aerated water and 300 g of sterilized sediments (weight ratio of sediments to water is 1:10) in the plastic containers, and the T2 group contained 3,000 ml of aerated water and 300 g of fresh sediment (weight ratio of sediments to water is 1:10) in the plastic containers (Figure 1A). Although the gauze packets did not allow burrowing or other behaviors in the sediment, the animals are accessible to the sediment in the packets due to the good permeability of the gauze. Treatments were conducted in the artificial climate chest with the same condition as those in acclimation. Experiments were started on 11 October 2019, and the treatments lasted for 20 days until 30 October 2019. Larvae were fed with sufficient red worm once a day (at 9:00 a.m.), while the sediments packets and water were replaced every 2 days (at 18:00).

### Sample Collection

After 20 days of treatment (Bletz et al., 2016), ten individuals from each treatment group were randomly selected. For each treatment group, the ten individuals were from four plastic containers, with 2–3 individuals being sampled from each plastic container to avoid the cage effect (animals from the same cage may share more symbiotic microbes due to exchange). The body weight of sampled individuals was measured to the nearest 0.01 g using a scale, and their body length was measured to the nearest 1 mm using a digital caliper. The skin, oral, stomach, and gut microbiota, as well as their brain, liver, spleen, and skin tissues, were collected. Details of the approaches are as follows: ultra-pure water was first used to rinse the larvae three times to remove potential transient bacteria. Sterile swabs were then used to collect the skin microbes by wiping the dorsal, ventral, and lateral sides of the larvae (Xu et al., 2020). For oral microbial sampling, sterile swabs were used by swabbing the oral cavity of the larvae gently and repeatedly. Later, larvae were euthanized with 0.1% MS-222 and dissected to collect the gut and stomach contents, which were preserved in 2 ml of the aseptic centrifuge tubes, separately. These samples were further used to do the gut and stomach microbial analyses, respectively.

### 16S rRNA Gene-Based Microbiome Analyses

The skin, oral, stomach, and gut microbes of two random individuals were pooled as one sample, and thus, we obtained five samples or biological replicates for each tissue and each group. The two individuals for pooled samples may be either from the same cage or not, as they were randomly selected. Additionally, 18 sediment samples were also prepared for analyses of bacterial diversity (*n* = 6 replicates for upstream, midstream, and downstream locations, respectively). The QIAamp DNA Stool Minikit (Qiagen, Valencia, CA, USA) was used to extract DNA from the samples at room temperature. The V4 region of the 16S rRNA gene was amplified with 515F (5 = -GTGCCAGCMGCCGCGGTAA-3=) and 806R (5 = -GACTACHVGGGTWTCTAAT-3=) primers (Caporaso et al., 2012). We used the following PCR thermocycling conditions: 95°C for 5 min, 35 cycles of 95°C for 30 s, 55°C for 30 s, and 72°C for 45 s, with a final extension step at 72°C for 10 min. High-throughput sequencing of amplicons was performed using the Illumina MiSeq platform. Sequencing was performed by Mingke Biotechnology Co., Ltd. (Hangzhou, China).

QIIME 1.9 was used to process the raw sequences and to obtain clean sequences (Caporaso et al., 2010). In the trimming analysis, *Usearch* was used for chimerism check in order to remove low-quality sequences (Edgar, 2010), *flash* program was used for splicing (Magoc and Salzberg, 2011), and *trimmomatic* program was used for quality control with default parameters (e.g., Window Size: 20 base pair; Minimum Read Length: 50 base pair) (Bolger et al., 2014). Operational taxonomic units (OTUs) were defined as sharing >97% sequence identity by annotating clean sequences to the SILVA132 database (Quast et al., 2013). The taxon summary was conducted using the OTU table in QIIME 1.9.

The alpha diversity (e.g., OTU richness and Shannon index) was calculated using QIIME 1.9 (Caporaso et al., 2010). A one-way ANOVA was used to investigate the differences in the microbial alpha diversity in each type of symbiotic microbiome between groups. The dissimilarity matrices (Bray-Curtis distances and unweighted and weighted UniFrac distances) were produced by the QIIME pipeline. The Bray-Curtis distances only considered the abundances of each OTU but not the phylogenetic relationships between bacterial taxa. The unweighted Unifrac and weighted Unifrac distances took the phylogenetic relationships into consideration. The difference is that the weighted Unifrac distances considered the abundance of each OTU, while the unweighted Unifrac distances only considered the presence or absence of each OTU in samples. In this study, the weighted Unifrac distances were preferentially used for comparative analyses, but analyses based on the other two types of distances were also provided in Supplementary Materials. Principal coordinates analysis (PCoA, based on dissimilarity matrices) and hierarchical clustering were used to visualize the dissimilarity of beta diversity, and PERMANOVA (Adonis function in the Vegan package) was performed to test the composition differences at the OTU level (Dixon, 2003). Benjamini–Hochberg (BH) correction was conducted to obtain the corrected *p*-values. The differences in microbial compositions between groups were compared using the linear discriminant analysis (LDA) effect size (LEfSe) method (Segata et al., 2011).

Source-Tracker 0.9.5 was used to assess the contribution (microbiome transmission) of the sediment and initial symbiotic microbiota (before treatment) to the building of the symbiotic microbiotas in treated larvae (Knights et al., 2011). Since the control group faced no variation in environmental conditions, we speculated that they kept the initial state of the symbiotic microbiota. For example, to explore the source of the oral microbiota of T1, the microbiotas of T1 individuals were set as sinks, while the sediment microbiotas and the skin, oral, stomach, and gut microbiotas of Cnt individuals were set as sources.

To evaluate the potential antifungal capacity of the microbiota, the 16S rRNA sequence of the OTUs was queried against an antifungal bacterial 16S rRNA database (Woodhams et al., 2015). Comparison hits with sequence similarity >95% were considered as valid one, and these OTUs were screened as antifungal strains and calculated for relative abundance (Xu et al., 2020).

### Transcriptomic Analyses

For each treatment group, the tissue samples of three individuals were randomly selected for transcriptomic analyses, and thus, there were three biological replications for each tissue and each group. The protocols of RNA extraction, purification, and cDNA library construction were used following the description from Chang et al. (2021). After cluster generation, the library preparations were sequenced on an Illumina HiSeq 4000 platform by Annoroad (Beijing), and paired-end reads were generated. The read quality was verified using the FastQC (version 0.10.0) software. Poly-N and low-quality reads, as well as those containing adapters, were removed. The total clean reads from all libraries were assembled *de novo* using Trinity as a reference transcriptome (refer to the assembly summary in Supplementary Table 1). The resulting unigenes were annotated by querying databases NCBI Non-Redundant Protein and Nucleotide Sequence databases (NR and NT databases), and Swiss-Prot with an *E*-value threshold of 1.0E-5, gene ontology (GO) with an *E*-value threshold of 1.0E-6, and Kyoto Encyclopedia of Genes and Genomes (KEGG) with an *E*-value threshold of 1.0E-10. The sequencing data from this study have been submitted to the Genome Sequence Archive (GSA; http://bigd.big.ac.cn/gsa/s/B7y0wob0) under accession number PRJCA004234.

Differently expressed genes (DEGs) were identified by DESeq (based on R) based on pairwise comparison (corrected *p* < 0.05, BH correction), or by one-way ANOVA across three groups (*p* < 0.01 or 0.05). Functional enrichment analyses were conducted by querying DEGs against the KEGG database (based on KOBAS 3.0, with default parameters) (Xie et al., 2011).

### Statistical Analyses

Basic statistical analyses were conducted using IBM SPSS 22.0 (SPSS Inc., Chicago, USA). Heatmaps were created by heatmap.2 in “gplot” package based on R software. The columns (samples) of the heatmap were not reordered, while the rows (genes) were reordered in default parameters. Graphs were drawn using Graphpad prism 5 or ggplot2, an R package (Wickham, 2009).

## Results

The sediment and host microflora are different in the bacterial community structure (Figure 1B, Supplementary Figure 2). There are 189/390 families and 438/830 genera exclusively existing in the sediment microflora (Figures 1C,D). Before treatment, the body length of the salamanders was 17.13 ± 0.25 cm (mean ± SE), and their body weight was 23.79 ± 0.87 g (mean ± SE). After 20 days of treatment, the body lengths of Cnt, T1, and T2 were mean = 18.12 cm ± 0.45 (SD), 17.68 cm ± 0.53 (SD), and 18.53 cm ± 0.48 (SD), respectively. Individual body weights of Cnt, T1, and T2 were mean = 31.23 g ± 1.66 (SD), 30.51 g ± 2.83 (SD), and 33.68 g ± 2.54 (SD), respectively. No significant difference in salamander growth rate was observed between groups (*p* > 0.05, one-way ANOVA; Supplementary Figure 3).

### Variations in Symbiotic Microbiota After Pre-exposure

**Figure 2:**
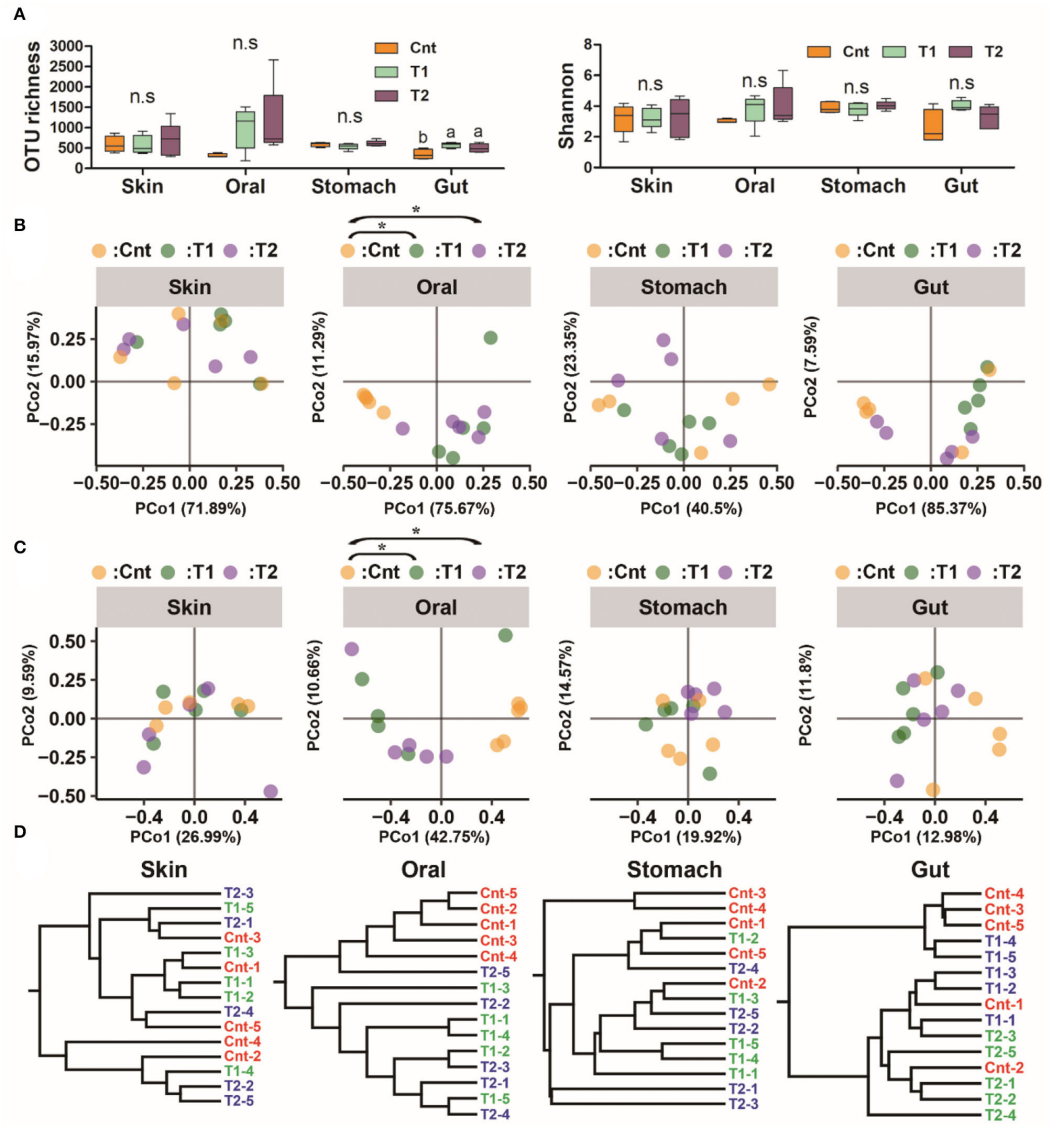
Influence of pre-exposure on the symbiotic microbiota diversity of giant salamander. **(A)** Variation of microbiome alpha diversity (left: operational taxonomic units (OTU) number; right: Shannon index). Data are presented as means ± SE (*n* = 5). The influences of pre-exposure on alpha diversity were tested using a one-way ANOVA and the Benjamini–Hochberg (BH) correction at the level of corrected *p* < 0.05. Different superscripts denote significant differences between groups (LSD *post-hoc* tests at *p* < 0.05) if there is a significant influence. **(B,C)** PCoA scatter plots present the dissimilarity of microbiomes at the OTU level [**(B)**, based on weighted UniFrac distance; **(C)**, based on unweighted UniFrac distance]. *Corrected *p* < 0.05, PERMANOVA followed by BH correction. **(D)** Hierarchical clustering based on weighted UniFrac distance.

The OTU richness in the gut microbiota was higher in T1 and T2 than in the Cnt group, while there were no intergroup differences in Shannon diversity of the gut microbiota (at corrected *p* < 0.05, one-way ANOVA, and BH correction; Figure 2A). No intergroup variations in alpha-diversity were observed for oral, skin, and stomach microbiota (at corrected *p* < 0.05, one-way ANOVA, and BH correction; Figure 2A).

**Figure 3:**
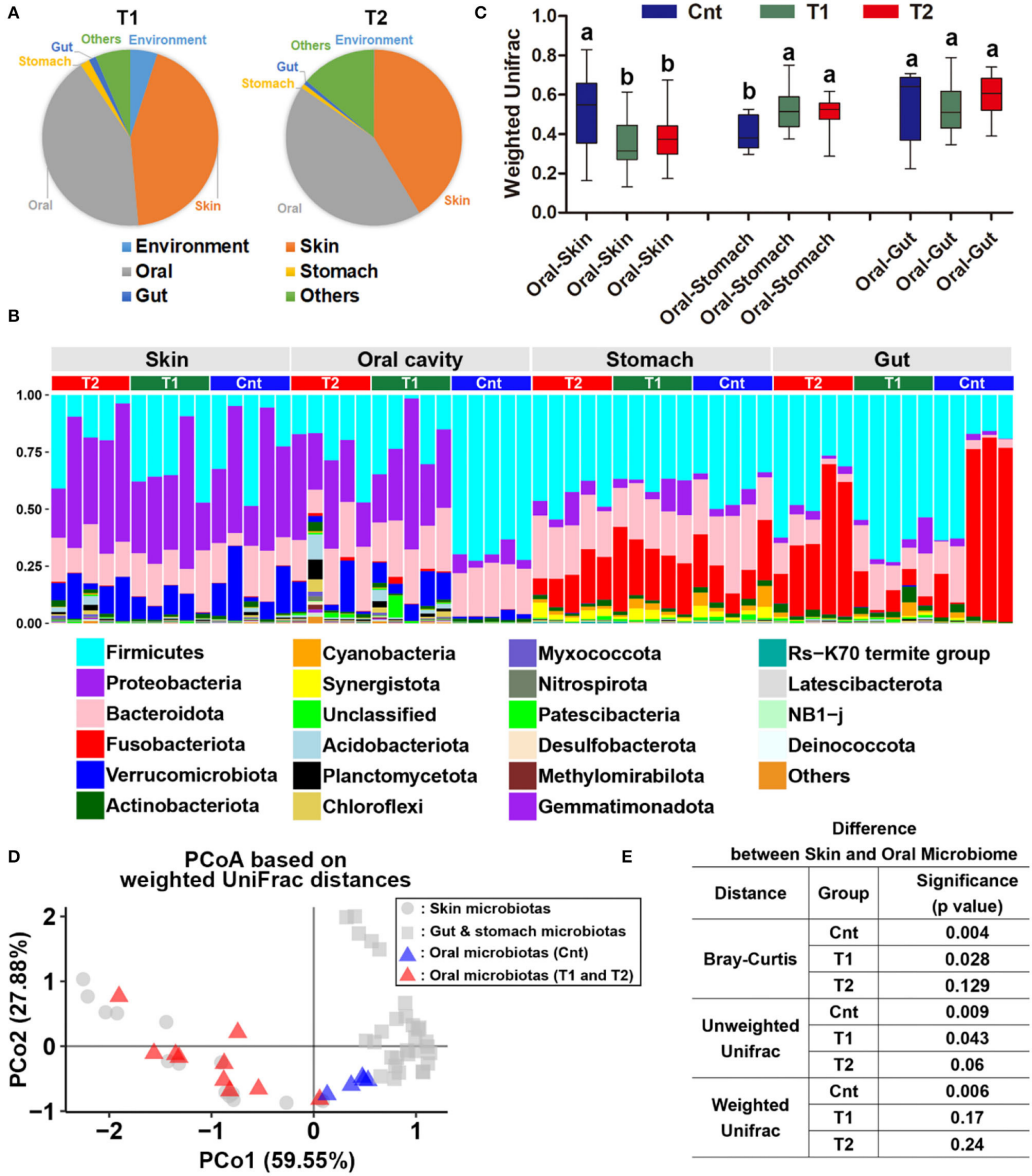
Reorganization of the oral bacterial community. **(A)** Source-tracking analyses for the oral microbiota of larvae in treatment groups. The habitat/environment and original commensal microbiota were considered as the bacteria sources. Since the control group faced no variation of environmental conditions during treatment duration, their commensal microbiota was considered the original commensal microbiota of captive *Andrias davidianus*. **(B)** Variation of weighted UniFrac distances between oral and other commensal microbiota after treatment. Data are presented as means ± SE. Different superscripts denote significant differences between groups (*p* < 0.05, one-way ANOVA and LSD *post-hoc* tests). **(C)** Bar plots represent the variation of microbiome similarity (at phylum level) between control, T1, and T2 groups. **(D)** A PCoA scatter plot presenting the variations of oral microbiota in the similarity to the skin, gut, and stomach microbiotas (Weighted UniFrac distance). **(E)** Results of PERMANOVA.

There was a significant difference in the larvae oral bacterial composition between Cnt and T1 groups (*R*^2^ = 0.60, corrected *p* = 0.039 for unweighted UniFrac distance; *R*^2^ = 0.56, corrected *p* = 0.015 for weighted UniFrac distance), as well as that between Cnt and T2 groups (*R*^2^ = 0.60, corrected *p* = 0.024 for unweighted UniFrac distance; *R*^2^ = 0.43, corrected *p* = 0.015 for weighted UniFrac distance) (PEMANOVA and BH correction). The oral samples could be divided into control and treatment groups (T1 & T2) by PCoA and Hcluster based on both weighted and unweighted UniFrac distances (Figures 2B–D). No significant differences in microbiota composition in the skin, stomach, or gut were observed between larvae from the control and treated groups (Cnt vs. T1 and Cnt vs. T2) (corrected *p* > 0.05, PERMANOVA and BH correction). “Source-track” analyses indicated that the original skin and oral microbiomes were the major sources of the oral microbiomes from both T1 and T2 larvae, while the habitat sediment microbiota contributed less (Figure 3A). Correspondingly, the individual oral microbiome (at phylum level) was more similar to the skin microbiome in the presence of sediment (Figure 3B). The weighted UniFrac distance between oral and gut microflora decreased significantly, while that between oral and stomach increased significantly (*p* < 0.05, one-way ANOVA, Figure 3C). PCoA and PERMANOVA of weighted UniFrac distance further indicated that the oral and skin microbiota showed increased similarity in the presence of wild sediment (Figures 3D,E).

**Figure 4:**
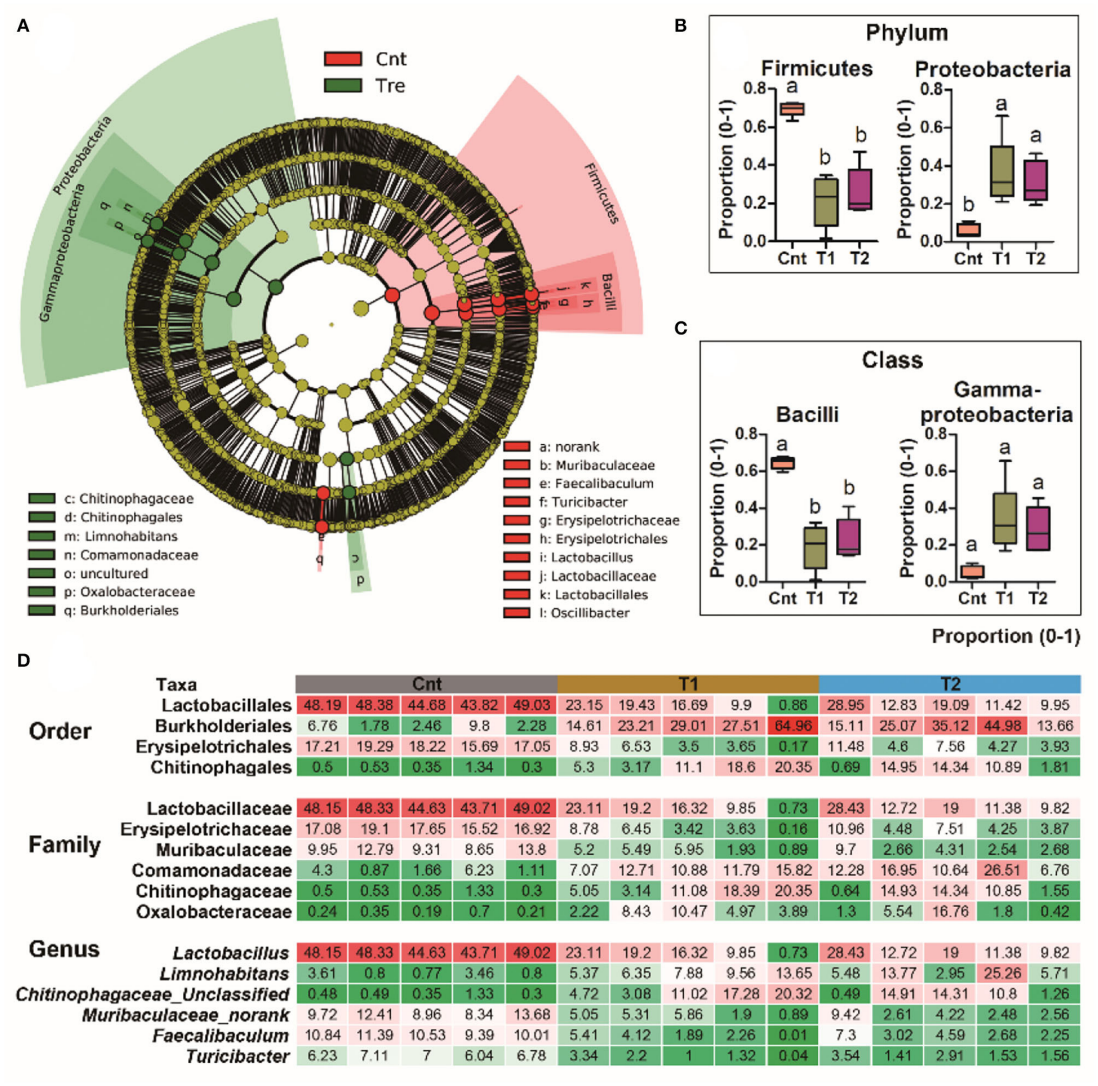
Differential analyses of the oral microbiota between control and treatment groups. **(A)** A LEfSe analysis on the oral bacteria composition between control and treatment groups. **(B,C)** The primary variations at phylum and class levels. Data are presented as boxplots (*n* = 5). Different superscripts denote significant differences between groups (*p* < 0.05, one-way ANOVA and LSD *post-hoc* tests). **(D)** Heatmap presents the major variations of microbiota at order, family, and genus levels. The values denote the abundances in percentages.

**Figure 5:**
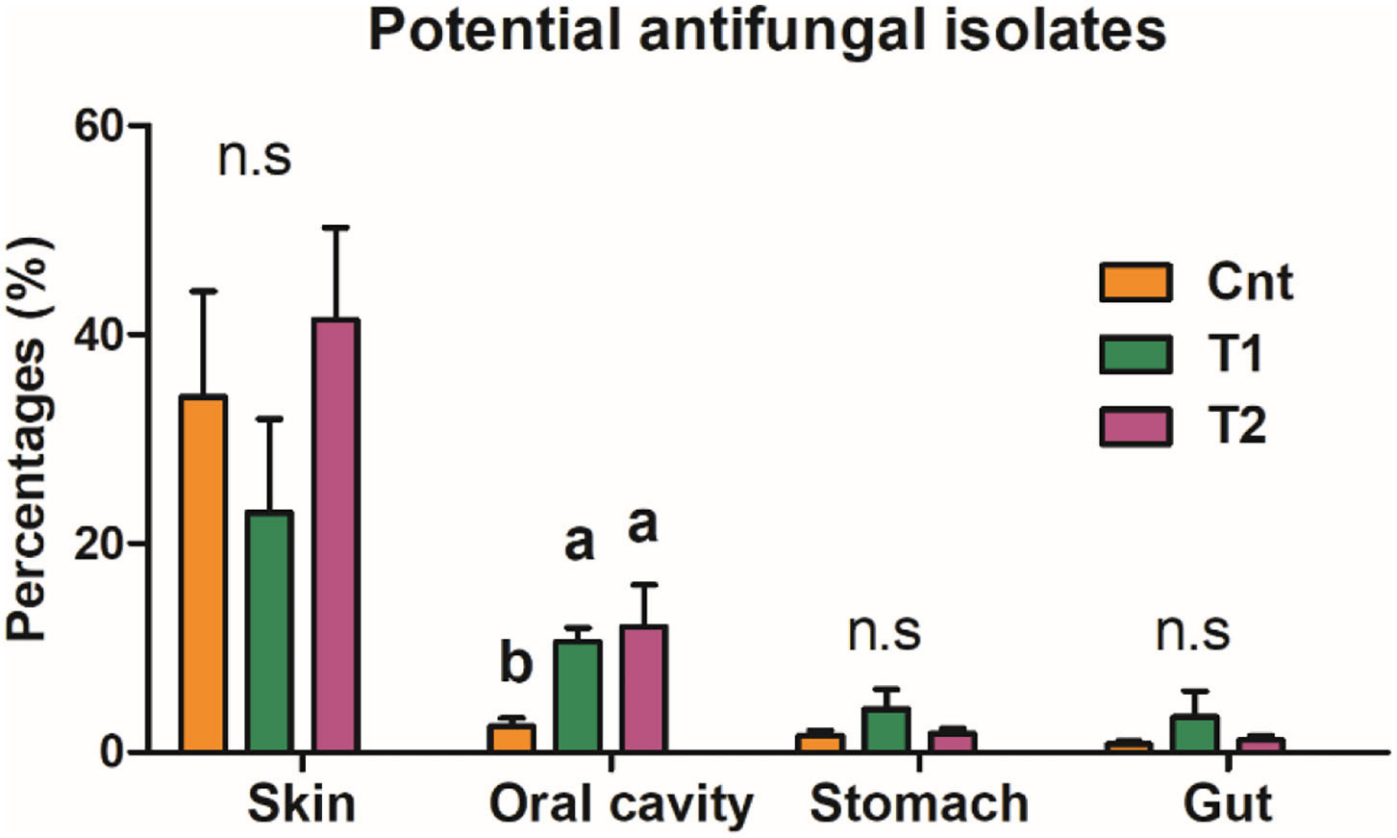
Proportion of antifungal OTUs in commensal microbiota. The influences of pre-exposure on the abundance of antifungal isolates were tested by a one-way ANOVA and the BH correction at the level of corrected *p* < 0.05. Different superscripts denote significant differences between groups (LSD *post-hoc* tests at *p* < 0.05) if there is a significant influence.

The oral bacterial community of T1 and T2 groups was characterized by the increase of Proteobacteria while the decrease of Firmicutes (Figures 4A,B). The decrease of Firmicutes was mainly due to the reduced abundance of Bacilli at the class level, Lactobacillales at the order level, Lactobacillaceae at the family level, and *Lactobacillus* at the genus level (Figures 4C,D). The increase of Proteobacteria was mainly contributed by gamma-Proteobacteria at the class level, Burkholderiales and Chitinophagales at the order level, and Comamonadaceae, Chitinophagaceae, and Oxalobacteraceae at the family level (Figures 4C,D). These variations were accompanied by a significant increase in antifungal OTU proportions in the oral microbiota (corrected *p* < 0.05, one-way ANOVA and BH correction; Figure 5).

### Transcriptional Changes of the Animal After Pre-exposure

**Figure 6:**
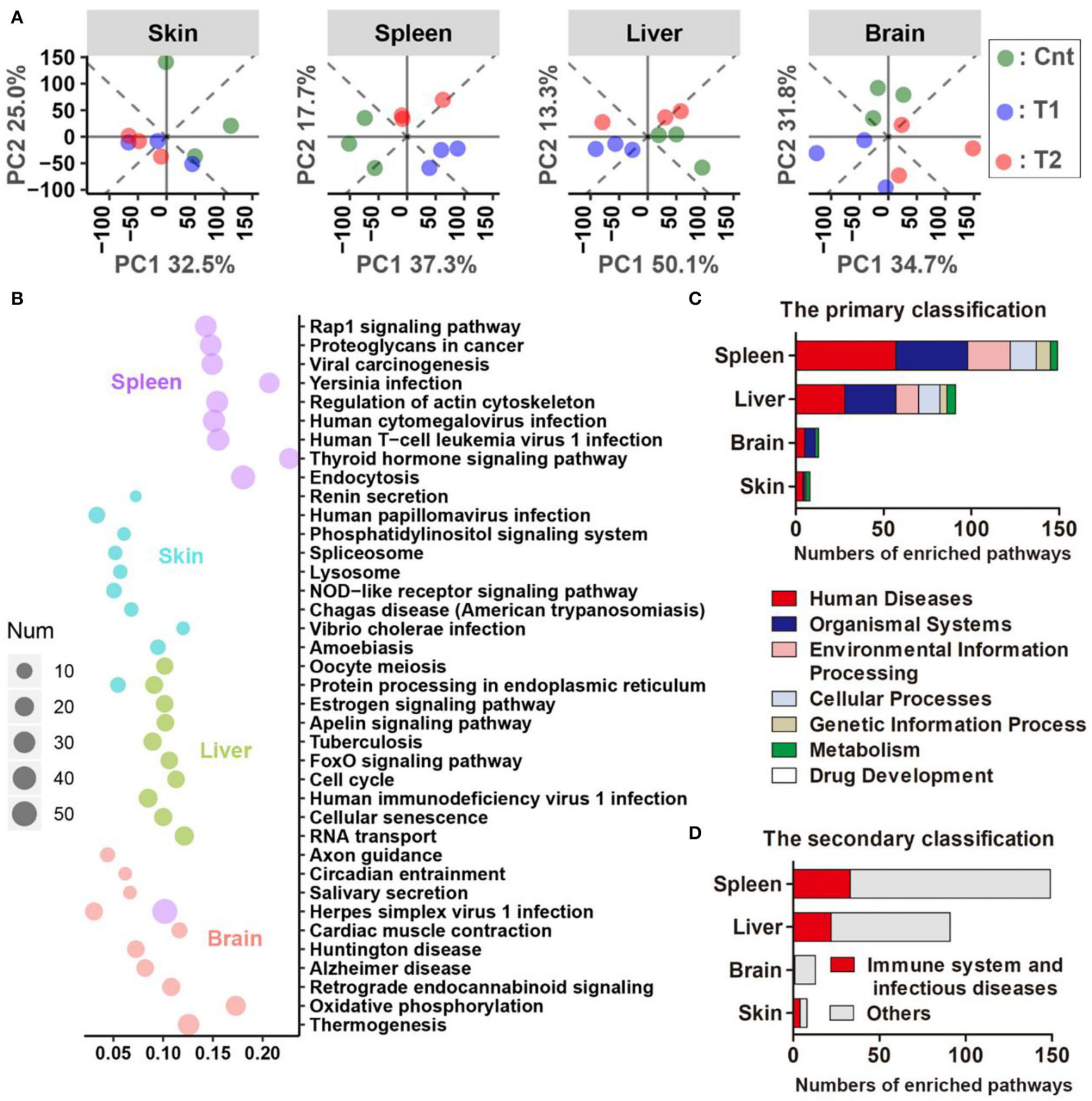
Results of comparative transcriptomic analyses. **(A)** Principal component analysis (PCA) scatter plots present the dissimilarity of the transcriptional profile between groups. **(B)** The top 10 Kyoto Encyclopedia of Genes and Genomes (KEGG) pathways (ordered by *q*-value) of each organ are enriched by the differently expressed genes (DEGs) (*p* < 0.01, one-way ANOVA). The size of each dot denotes the number of DEGs enriched in each pathway. The horizontal axis stands for the enrichment cover rate of each pathway (enriched gene number/total gene number of this pathway). It should be noted that many immune pathways in the KEGG database are named after the pathogens, but they may share considerable immune proteins in their signal transduction networks. For example, the highlight of the Herpes simplex virus 1 pathway might not mean the infection of this virus in the giant salamander, but it emphasizes that there were a lot of immune-related genes that varied after pre-exposure. **(C,D)** Statistics on the functional category of all the significantly enriched pathways (corrected *p* < 0.05). KEGG pathways are classified into seven categories at the primary classification level. Each primary category is further divided into secondary classes according to its cellular function (https://www.kegg.jp/kegg/pathway.html).

For the pairwise comparison between groups, there were more DEGs (corrected *p* < 0.05, *T*-test, and BH correction) between the control and treatment groups than those between the T1 and T2 groups for all four organs (Supplementary Figure 4A). The four organs shared few DEGs with each other, and the spleen showed the most dramatic variations in their transcriptomes (Supplementary Figures 4B,C). The cnt individuals could be distinguished from sediment-treated ones by the skin transcriptomes, and the Cnt, T1, and T2 individuals could be divided into their respective groups by the spleen, liver, or brain transcriptomes [principal component analysis (PCA) analyses, Figure 6A]. Functional enrichment analyses were conducted for DEGs (*p* < 0.01, one-way ANOVA) across Cnt, T1, and T2 groups. The top 10 enriched pathways (sorted by corrected *p*-value, KOBAS 3.0) of each organ were presented to show the primary transcriptional variations (Figure 6B). In the spleen, these pathways were mainly involved in immune response and infectious diseases (e.g., herpes simplex virus 1 infection, human T-cell leukemia virus 1 infection, and endocytosis). In the skin, most of these pathways were also related to immune responses and infectious diseases, including amoebiasis and the NOD-like receptor signaling pathway. Moreover, protein metabolic pathways were also highlighted, such as protein processing in ER and lysosome. In the liver, the top 10 enriched pathways were diverse in function, covering cellular proliferation (e.g., cell cycle and oocyte meiosis) and numerous signaling transduction (e.g., FoxO signaling pathway and Apelin signaling pathways). However, immune-related pathways (i.e., tuberculosis and human immunodeficiency virus 1 infection) were still involved. In the brain, oxidative phosphorylation (OPP) was highlighted. In addition, OPP enzymes were also responsible for the enrichment of many other pathways (e.g., thermogenesis, Alzheimer's disease, Huntington's disease, and cardiac muscle contraction). Moreover, functional enrichment analyses also highlighted many immune-and disease-related pathways (e.g., human papillomavirus infection, chemokine signaling, and Fc epsilon RI signaling pathway) (Supplementary Figure 5). Further analyses classified the significantly enriched pathways (corrected *p* < 0.05) into functional categories defined in the KEGG database. At the primary classification level, the “Human diseases” category accounted for the largest number of enriched pathways in the spleen, liver, and skin (Figure 6C). At the secondary classification level, “Immune system” and “Infectious diseases” occupied large proportions in these three organs (Figure 6D).

**Figure 7:**
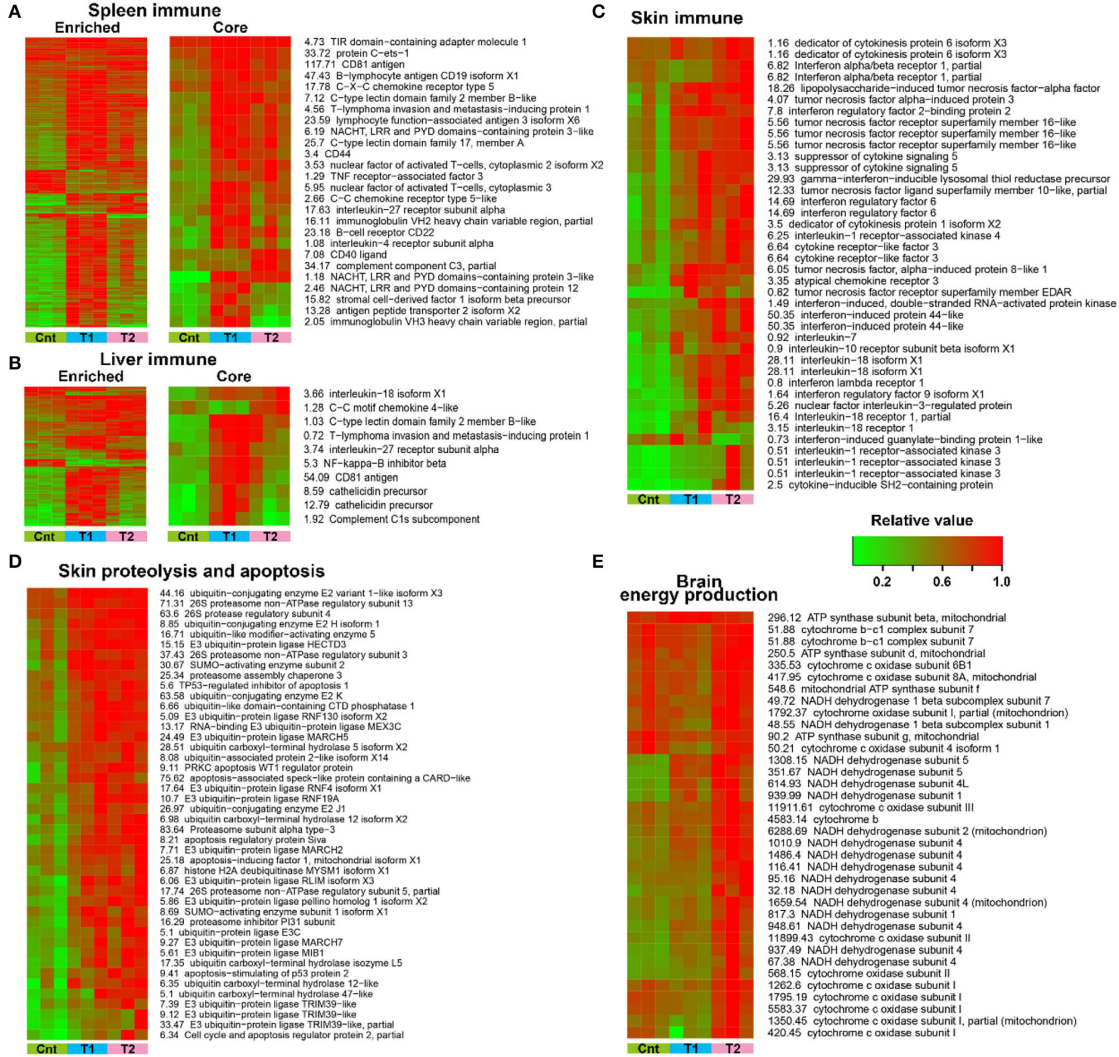
Transcriptional variation trends of crucial and significantly enriched cellular processes. **(A,B)** Expression pattern of DEGs enriched in immune-related pathways in the spleen **(A)** and liver **(B)**. The core immune genes refer to those with defined immune functions. **(C–E)** Expression pattern of inflammation-related DEGs in the skin **(C)**, proteolysis and apoptosis-related DEGs in the skin **(D)**, and energy metabolic DEGs in the brain **(E)**.

The variation trends were presented for DEGs (*p* < 0.01, one-way ANOVA) with closely related functions. In the spleen and liver, DEGs enriched in immune-related pathways showed an overall upregulation in treated larvae, and the T1 group showed more prominent upregulation than the T2 group (Figures 7A,B). This variation pattern was still presented when focused on DEGs with definite immune functions (e.g., interleukin-18, cathelicidin, and immunoglobulin; screened manually) (Figures 7A,B). In the skin, the treatment caused an overall upregulation of immune (most identified inflammatory cytokines) and proteolytic and apoptotic components (e.g., most of the identified ubiquitination components and apoptosis-inducing factors) (Figures 7C,D). No significant difference existed in the transcriptional level of these genes between the T1 and T2 groups. In the brain, the OPP enzymes showed prominent upregulation in the T1 group, but not in the T2 group (Figure 7E). In addition, the differently expressed immune-related genes (e.g., MHC, pattern recognition proteins, and pro-inflammatory factors) were screened, and most of these genes in the liver, spleen, and skin showed increased transcription after pre-treatment (Supplementary Figure 6).

## Discussion

### Compositional and Functional Shift of Symbiotic Microbiome

Our results provided a detailed description of the community structure of multiple symbiotic microbiotas in captive *A. davidianus*. Consistent with our previous studies (Zhang et al., 2019; Zhu et al., 2021), Firmicutes, Bacteroidetes, Fusobacteria, and Proteobacteria were the dominating bacteria in the stomach and gut microbiome of *A. davidianus*. During pre-exposure, a large amount of new bacterial taxa can be brought to the living environments of captive individuals (Figure 1), providing opportunities for these new microbes to colonize the skin and digestive tract of the host (i.e., captive *A. davidianus* larvae). However, no significant change in the bacterial community was observed in the skin, stomach, and gut after the treatment (Figure 2). It suggested that the symbiotic microflora in the skin, stomach, and gut of captive individuals is relatively stable. The skin microbiome of amphibians has been proved to play an important role in their innate immunity (Figure 5; Woodhams et al., 2007; Colombo et al., 2015; Varga et al., 2019; Douglas et al., 2020), and the gut microbiome is responsible for maintaining gut metabolic homeostasis and coordinating immune responses (Kau et al., 2011; Shapira, 2016). Their stability may benefit the host to prevent the colonization of pathogens (Lauer et al., 2008; Harris et al., 2009; Kung et al., 2014).

Unlike the situation for the skin, stomach, and gut, the oral microflora showed prominent reorganization in treated individuals. It seemed that the oral microbiome was more flexible in composition than that of the skin, stomach, and gut. The oral cavity is a transitional region linking the external environment and the intestinal space. Oral microbiota is affected by diet, age, and environmental factors, and synergy and interaction of variable oral microorganisms can help the host against the invasion of undesirable stimulation outside (Gao et al., 2018). In humans, the variation of oral microbiota is associated with oral diseases (e.g., caries and mucosal diseases) and systemic diseases (e.g., diabetes and obesity) (Gao et al., 2018; Mameli et al., 2019; Frias-Lopez and Duran-Pinedo, 2020). This is suggestive of the immune and metabolism-regulatory functions of the oral microbiota. Pre-exposure to the soil sediment caused the dominating oral bacteria to change from Firmicutes to Proteobacteria (Figure 2). A high proportion of Firmicutes is associated with increased nutrient absorption and energy storage in mammals (Turnbaugh et al., 2006; Krajmalnik-Brown et al., 2015; Dugas et al., 2016). In addition, it has been reported that there is an association between obesity and increased abundance of Firmicutes in oral biofilm in humans (Zeigler et al., 2012). Specifically, the reduction of Firmicutes and Bacilli in the oral cavity was largely contributed by the loss of Lactobacillus, which has been used as probiotics to promote growth and survival in the breeding industry (Venkat et al., 2004). Taken together, the oral microbiota of the captive individuals seemed to be in favor of host nutrient absorption and growth. This might be a result of long-term adaptation to an artificial environment, where animal growth rate and body weight were pursued. In contrast, Proteobacteria is a typical dominating phylum in the skin microbiota of amphibians (Catenazzi et al., 2018), which is related to amphibian defense and immunity (Woodhams et al., 2007; Colombo et al., 2015; Varga et al., 2019; Douglas et al., 2020). The increased proportion of Proteobacteria in the oral microbiome was largely contributed by the raise of gamma-Proteobacteria. This class contains higher proportions of anti-fungi strains than others (Catenazzi et al., 2018; Rebollar et al., 2019, 2020). In fact, the abundance of anti-fungus strains indeed increased after pre-exposure. Thus, we might expect increased capacity in fungal pathogen defense of the pre-adapted oral microbiome. Such a structural shift in oral microbiota might be beneficial to coping with the complicated microbial community of the natural habitats.

Importantly, this change was not contributed by the introduction of exogenic microorganisms (Figure 3A). This was supported by the fact that the changes in oral microbiota were observed no matter whether the soil sediment was sterilized or not. The previous study has indicated that the oral and skin microbiomes of amphibians and captive Komodo dragons are strongly affected by the environment (Hyde et al., 2016; Xu et al., 2020). One of the explanations for our observation was that prolonged treatment was required to allow the colonization of the environmental microbiota to the captive *A. davidianus*, especially when considering the low dosage of the sediment introduced into the experimental system. Further pre-exposure studies should take the developmental stage or age into consideration, which can shape the gut microbiota of *A. davidianus* (Zhang et al., 2019), as it is possible for variation in the plasticity of symbiotic microbiota with age in response to the environment. Our results suggested a significant contribution of skin microbiota to the change of oral microbiota when exposed to the habitat sediment. This was reasonable because there is a robust exchange between skin and oral microbiota in animals (Buerger, 2020). The easy transmission of microbes from the skin to oral microbiota may be explained by the “old friends” hypothesis (Rook, 2013), which postulates that the members of the symbiotic microbiome would be identified as non-pathogenic by the immune system during an exchange event.

### The Transcriptional Changes of Immune and Nervous Systems in the Host

**Figure 8:**
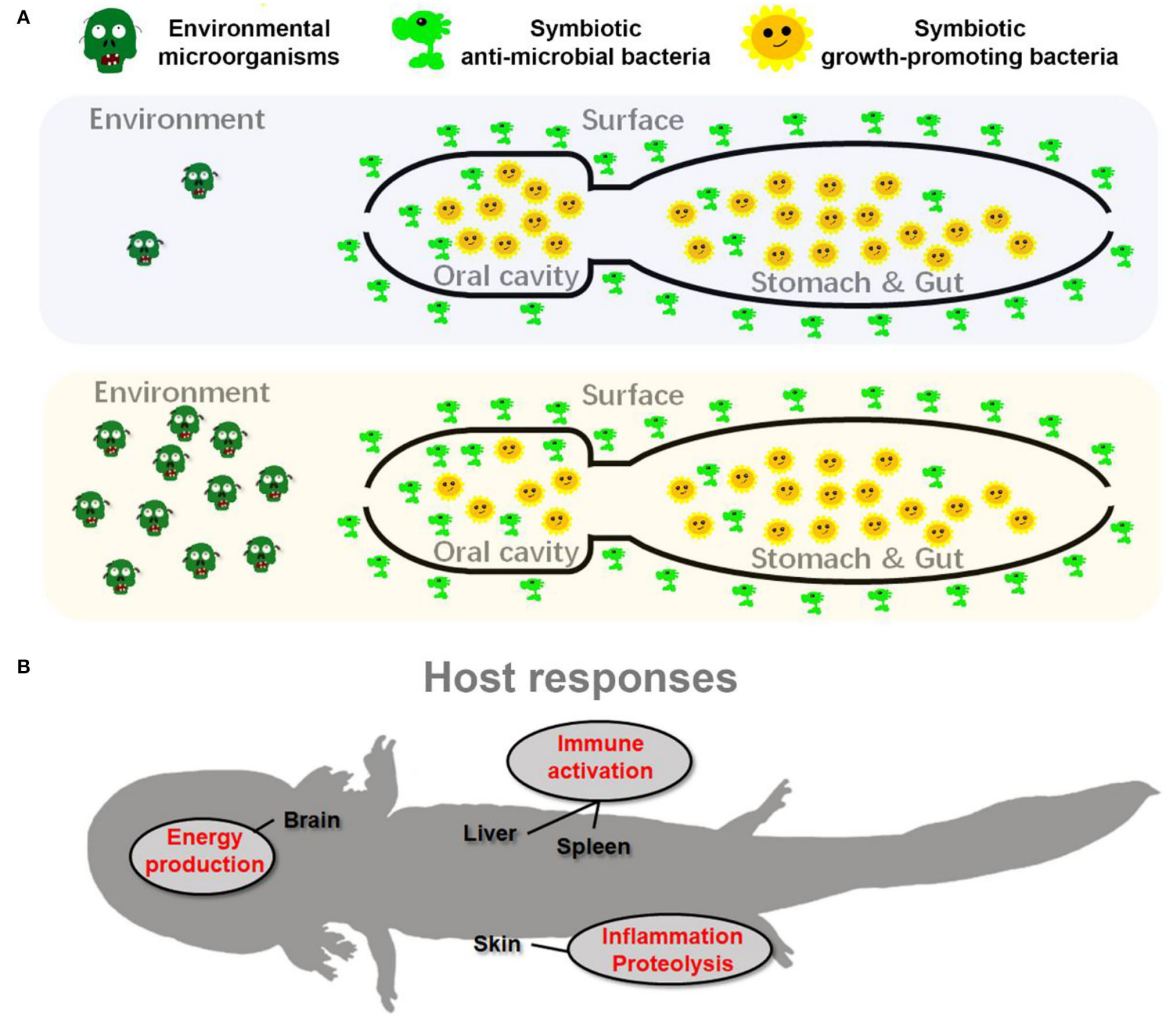
Schematic diagram presents the adaptive adjustments of giant salamander to habitat river sediment acclimation. **(A)** The adjustments of symbiotic microflora; **(B)** the host transcriptional responses.

Our data provided an insight into the transcriptional architecture of multiple organs of *A. davidianus*. Pre-exposure to habitat sediments caused dramatic transcriptional variations in the brain, liver, spleen, and skin of captive *A. davidianus* (Figure 6A). In the skin, the variations of genes were characterized by the upregulation of inflammatory systems (Figure 7C, Supplementary Figure 6), indicating the possibility of pathogenic stimulus (Akira et al., 2006; Chen et al., 2018). Meanwhile, the ubiquitin-dependent proteolysis and apoptosis were transcriptionally activated (Figure 7D), suggesting the accelerated turnover of skin tissue. It might be an approach to void exogenous microorganism colonization by promoting antigen recognition and pathogen elimination (Goldberg and Rock, 1992; Miyairi and Byrne, 2006; Sasakawa and Hacker, 2006). Further validation was necessary to verify whether there was a causation relationship between transcriptional activation of inflammation and proteolysis. Existing studies indicate that dead pathogen stimulation can also enable a similar magnitude of acquired resistance for amphibian skin (Mcmahon et al., 2014). This might explain why similar transcriptional changes were also detected in the skin of T1 individuals, where the soil sediment was sterilized before treatment. The spleen is responsible for adaptive immune response through humoral and cell-mediated pathways in vertebrates (Mebius and Kraal, 2005). The upregulation of immune-related genes (Supplementary Figure 6) in the spleen likely suggested enhanced immune function in *A. davidianus* larvae after exposure to habitat sediment. It was interesting to detect a robust immune response in the spleen and the liver of individuals from the T1 group (Figures 7A–C). This might be attributed to the high temperature (during sterilization) that could break down the cellular structure of microorganisms, as well as the spatial structure of macromolecules, thus causing the exposure of hidden antigen epitopes, which would induce strong local immune stimulation (Cattoretti et al., 1993). Additionally, the killed microorganisms could be broken into small peptides or oligosaccharides with antigen epitopes by digestive enzymes. Additionally, these fragments might be transported into the host to activate systematic immune responses (such as the oral vaccines) (Brayden et al., 2005). In the brain, individuals from the T2 group showed transcriptional upregulation of OPP enzymes (Figure 7E), suggesting the improved activity of the central nervous system. Considering the absence of this phenomenon in T1 individuals, we guessed that this transcriptional change likely resulted from the stimulation of living organisms or heat-sensitive components from the sediments. It may be explained by the microbiota–gut–brain axis, which describes the influence of gut microbiota on the host neuroendocrine system by generating metabolic products or interacting with the immune system (Foster and Mcvey Neufeld, 2013; De Vadder et al., 2014; Yano et al., 2015; Sharon et al., 2016). The specific inducement for these transcriptional variations and the underlying mechanisms require further investigation. In addition, increased energy metabolism in the brain likely improves the capacity of information processing (e.g., foraging) to adapt to complicated environments (Lefebvre et al., 1997; Dunbar and Shultz, 2007). Overall, these results suggested that pre-exposure to habitat sediments might have beneficial effects on captive *A. davidianus* by pre-activating the immune systems and brain activity (Figure 8B).

### Implications for *A. davidianus* Reintroduction

Our results indicated that pre-exposure to habitat sediments could induce a series of physiological responses in captive *A. davidianus* (Figure 8). Although these adjustments might also be induced in larvae released to the field without pre-exposure, the expression degree of these adaptive traits might be limited. The major limitation may lie in the nutrient acquirement and energy budget. From the perspective of symbiotic microbiota, the reorganization of oral microflora may be related to the improved pathogen defense along aside from decreased nutrient absorption of these animals (Figure 8A). From the perspective of the host, maintaining brain metabolism and immune responses account for a large proportion of the body's energy requirement (Rolfe et al., 1997; Wang A. et al., 2019; Cunnane et al., 2020). Upregulation of the brain and immune activities during pre-exposure would constrain the energy budget in *A. davidianus* larvae. For larvae reintroduced to the field, we might not expect good nutrient status for their unskilled foraging and hunting at the beginning. Consequently, the nutrient status of the *A. davidianus* larvae might either limit the expression of these adaptive traits or impact the individual growth. Pre-exposure in an artificial environment where food can be provided sufficiently, which likely benefits their activation of adaptive responses. It can guarantee the investment in immune function and somatic growth simultaneously and thus improve their survival rate (Supplementary Figure 3). Therefore, we argued that pre-exposure to the field environmental microbiota can be included in the standard procedure of captive-bred *A. davidianus* larvae reintroduction. However, despite experimental approaches supporting the effectiveness of pre-exposure to the reorganization of symbiotic microbiota and host transcriptomes in captive-bred *A. davidianus* larvae, long-time field monitoring is still needed in the future to quantify its advantages in reintroduction activities.

## Data Availability Statement

The datasets presented in this study can be found in online repositories. The names of the repository/repositories and accession number(s) can be found at: http://bigd.big.ac.cn/gsa/s/B7y0wob0, PRJCA004234.

## Ethics Statement

The animal study was reviewed and approved by the Animal Ethical and Welfare Committee of Chengdu Institute of Biology, Chinese Academy of Science.

## Author Contributions

WeiZ, TZ, and JJ conceived the project. TZ, CZ, JC, JF, WenZ, FX, and CL collected the samples. CZ, WeiZ, JF, and WenZ performed the experiments. WeiZ, LC, and JL analyzed the data. WeiZ and TZ wrote the manuscript. All authors edited and approved the final version of the manuscript.

## Funding

This study was supported by the National Natural Science Foundation of China (31900327); the National Key Programme of Research and Development, Ministry of Science and Technology (2016YFC0503200); the Biodiversity Survey and Assessment Project of the Ministry of Ecology and Environment, China (2019HJ2096001006); the Construction of Basic Conditions Platform of Sichuan Science and Technology Department (2019JDPT0020); the China Biodiversity Observation Networks (Sino BON or Sino BON–Amphibian & Reptile).

## Conflict of Interest

The authors declare that the research was conducted in the absence of any commercial or financial relationships that could be construed as a potential conflict of interest.

## Publisher's Note

All claims expressed in this article are solely those of the authors and do not necessarily represent those of their affiliated organizations, or those of the publisher, the editors and the reviewers. Any product that may be evaluated in this article, or claim that may be made by its manufacturer, is not guaranteed or endorsed by the publisher.
