# Supplementary material for: Effects of Habitat River Microbiome on the Symbiotic Microbiota and Multi-Organ Gene Expression of Captive-Bred Chinese Giant Salamander
Source: Front Microbiol. 2022 Jun 13;13:884880. doi: 10.3389/fmicb.2022.884880 (PMC9234736; doi:10.3389/fmicb.2022.884880)
Supplement: Supplementary file 1 [file Data_Sheet_1.docx]

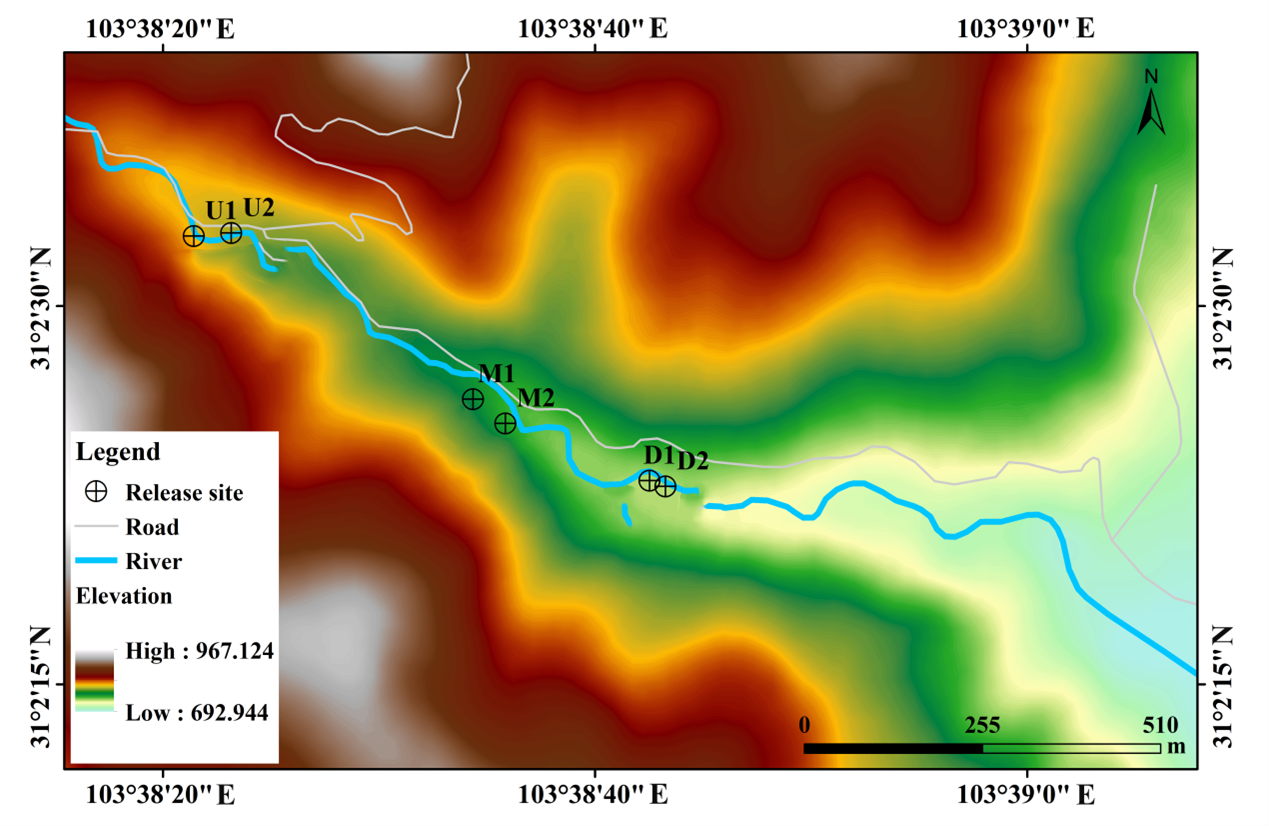


Figure S1 The river sediments sites collected in the present study. U1 & U2: upstream sites; M1 & M2: midstream sites; D1 & D2: downstream sites.


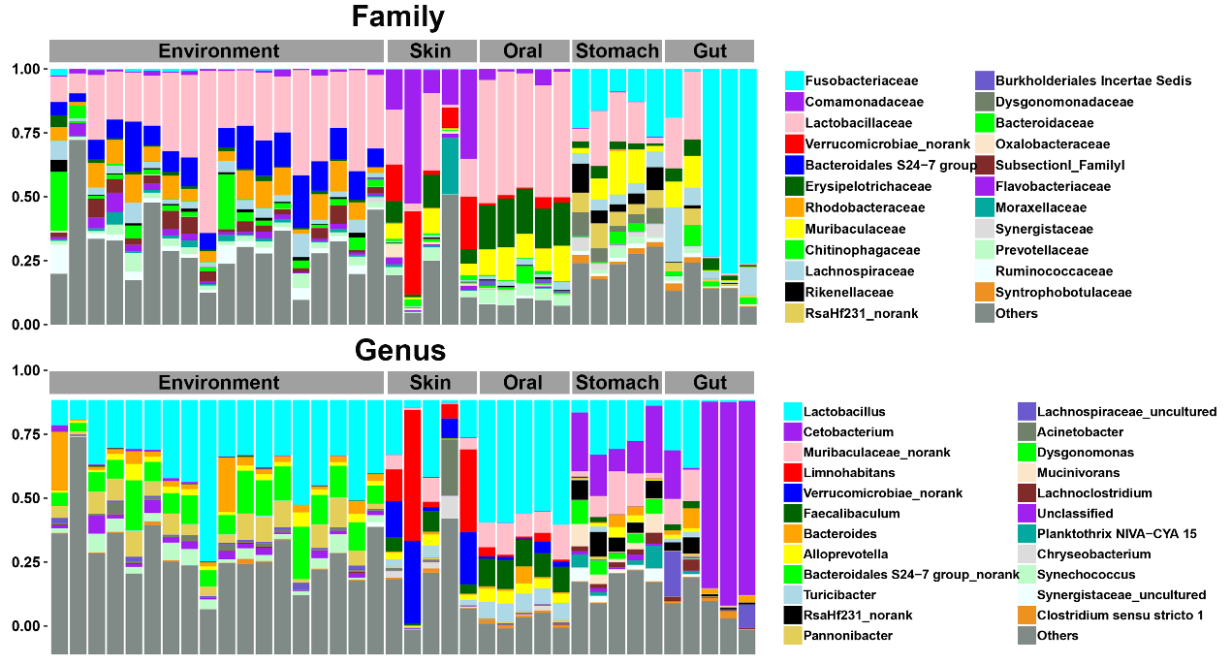


Figure S2 Soil and symbiotic microbiomes at family and genus levels.


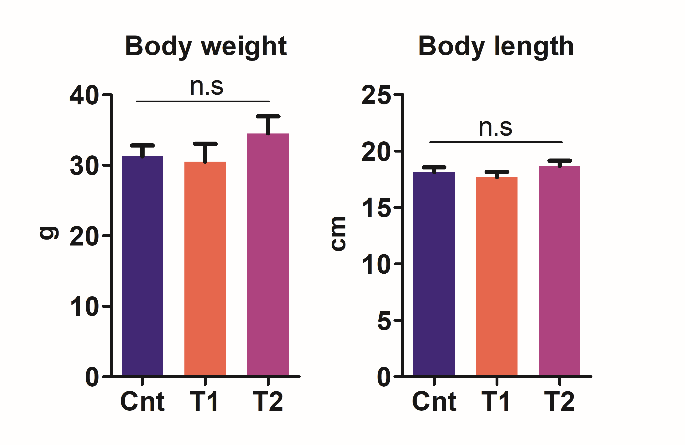


Figure S3 Larvae body traits before sampling collection. Data are presented as means ± SE (n = 10). The difference between groups was tested by one-way ANOVA followed by L.S.D *post hoc* test.


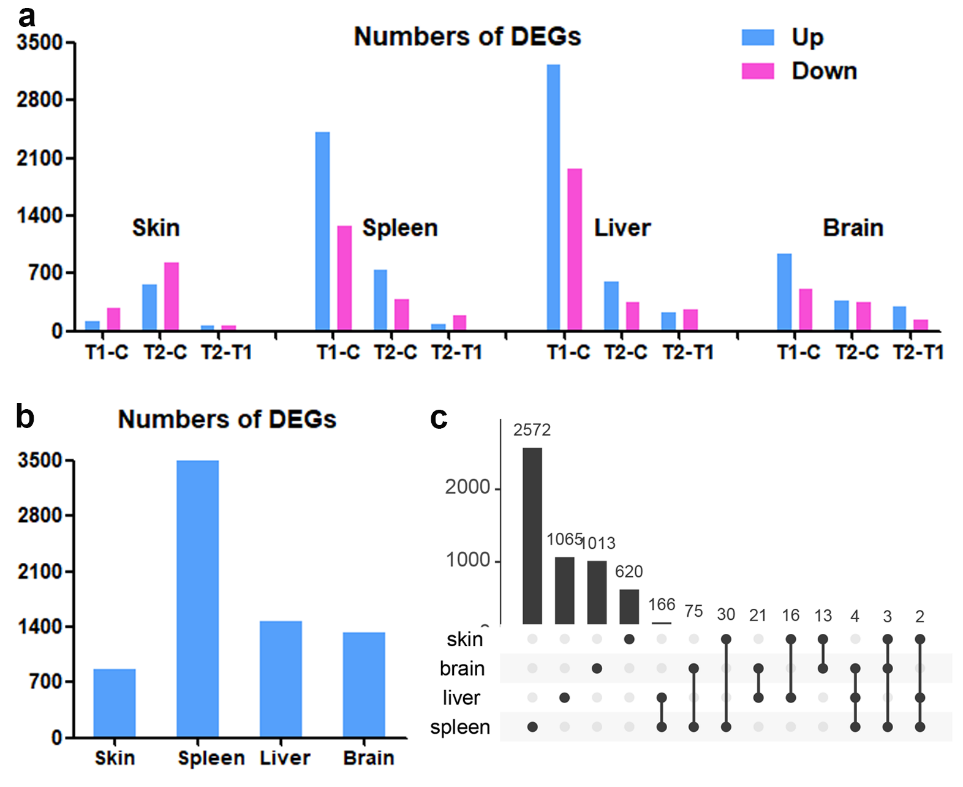


Figure S4 An overview of the transcriptional differences between groups. (a) Numbers of DEGs in pair wise comparison (corrected *p* < 0.05, DESeq and Benjamini and Hochberg’s correction). (b) Numbers of DEGs across the control, T1, and T2 groups (*p* < 0.01, one-way ANOVA). (c) The shared and unique DEGs between organs (*p* < 0.01, one-way ANOVA).


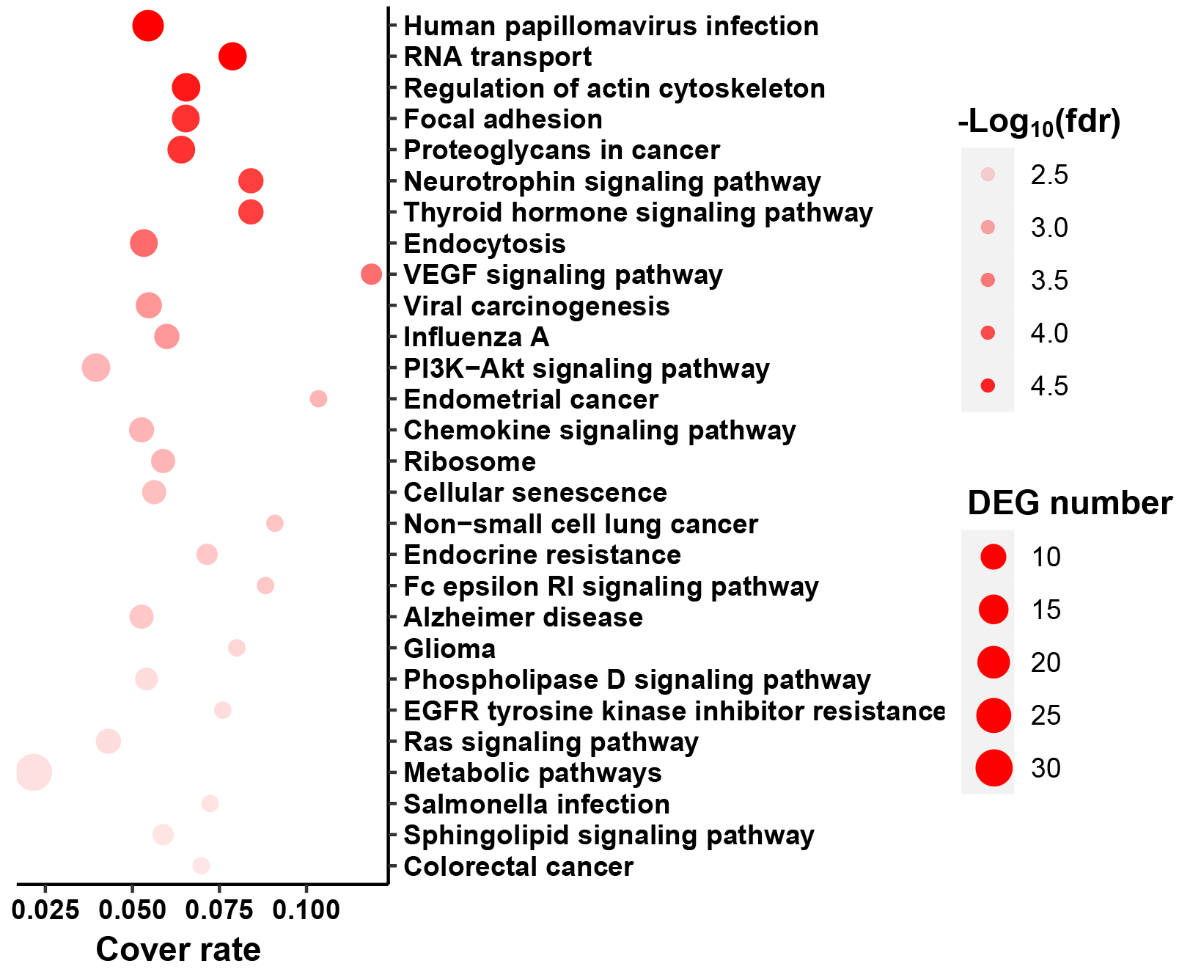


Figure S5 KEGG enrichment analyses based on DEGs (*p* < 0.05) shared by more than three organs. The KEGG items meeting the threshold of corrected *p* < 0.005 were presented.


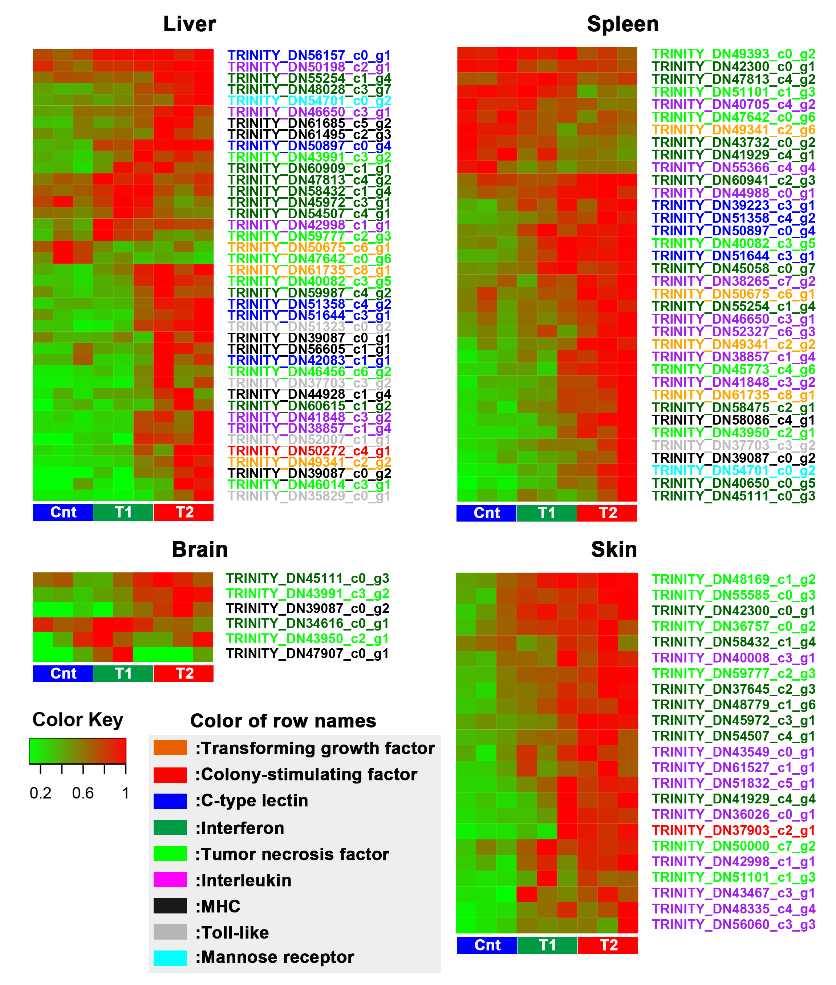


Figure S6 Heatmaps presenting the variations trends of significantly changed (*p* < 0.05, one-way ANOVA) immune-related genes (maximum FPKM > 5 across samples).

**Table S1** A summary of the transcriptome assembly.

| **Items** | **Trinity** | **Unigene** |
| --- | --- | --- |
| **Count** | 619,825 | 280,362 |
| **Percent GC (%)** | 47.14 | 46.61 |
| **Total Bases** | 520,275,393 | 206,533,118 |
| **N50** | 1,779 | 1,276 |
| **N90** | 297 | 280 |
| **Min** | 201 | 201 |
| **Max** | 53,437 | 53,437 |
| **Count** | 619,825 | 280,362 |
| **Mean** | 839.39 | 736.67 |
